# Step-by-step inpatient rehabilitation for critical illness after coronavirus disease 2019: A CARE-compliant case report

**DOI:** 10.1097/MD.0000000000026317

**Published:** 2021-06-11

**Authors:** Dae-Won Gwak, Jong-Moon Hwang

**Affiliations:** a Department of Rehabilitation Medicine, Kyungpook National University Hospital; b Department of Rehabilitation Medicine, School of Medicine, Kyungpook National University, Daegu, Republic of Korea.

**Keywords:** coronavirus disease 2019, critical illness, deconditioning, intensive care unit, rehabilitation

## Abstract

**Introduction:**

Since the coronavirus disease (COVID-19) outbreak in Wuhan, China, in December 2019, COVID-19 has become a worldwide pandemic. Muscle weakness and deconditioning caused by COVID-19-induced critical illness requires rehabilitation.

**Patient concerns:**

A 74-year-old male patient complained of general weakness after COVID-19, requiring ventilator treatment.

**Diagnosis:**

He was confirmed as having COVID-19 using a polymerase chain reaction test.

**Interventions:**

During admission in the intensive care unit, medical staff wearing level D protective equipment performed the bedside manual range of motion exercise. After a negative COVID-19 test, the patient was transferred to a general ward, where sitting balance training and pulmonary rehabilitation were additionally performed by rehabilitation therapists wearing protective gear. When the patient was able to stand up with support, standing balance training and sit-to-stand training were performed.

**Outcomes:**

After a month of rehabilitation, the patient could sit alone, but he needed help with standing balance. The Berg Balance Scale score improved from 0 to 4, and the Modified Barthel Index score improved from 8 to 18. He was able to breathe in room air without an oxygen supply.

**Lessons:**

This case report shows an example of how safe and effective rehabilitation can be provided to COVID-19 patients.

## 1 Introduction

Severe acute respiratory syndrome coronavirus 2 infection, later named as coronavirus disease (COVID-19) by the World Health Organization, became a worldwide pandemic since it was first detected in Wuhan, China, in December 2019.^[1,2]^ As of February 2021, more than 110 million cases and more than 2.4 million deaths have occurred worldwide since the epidemic began.^[3]^ The clinical variety of COVID-19 ranges from asymptomatic or mild disease to critical illness requiring treatment in intensive care unit (ICU) or death.^[4,5]^ Most cases of COVID-19 only present with mild symptoms, but some cases present with serious disease.^[6]^ Elderly patients in particular are more likely to develop serious illness after COVID-19, and the mortality rate of elderly patients with COVID-19 is higher than that of young or middle-aged patients.^[7]^

COVID-19-induced critical illness causes deconditioning, muscle weakness, or deterioration of function, and rehabilitation treatment is required.^[8]^ Although the need for rehabilitation treatment due to COVID-19-associated critical illness is increasing,^[9]^ severe acute respiratory syndrome coronavirus 2 is easily transmitted in confined spaces. Infection is more likely to spread during rehabilitation, including physical contact between the therapist and the patient.^[10]^ As a result, safe and effective rehabilitation methods for patients with COVID-19 have become an emerging topic.

Here, we present a case of rehabilitation after COVID-19-induced critical illness. We share our experiences of effective and safe rehabilitation treatment for improving physical function in patients with COVID-19. This case report was approved by the institutional review board of Kyungpook National University Hospital (2020-12-032-001). And, the written consent was obtained from the patient for publication of case details and images.

## 2 Case presentation

**Figure 1:**
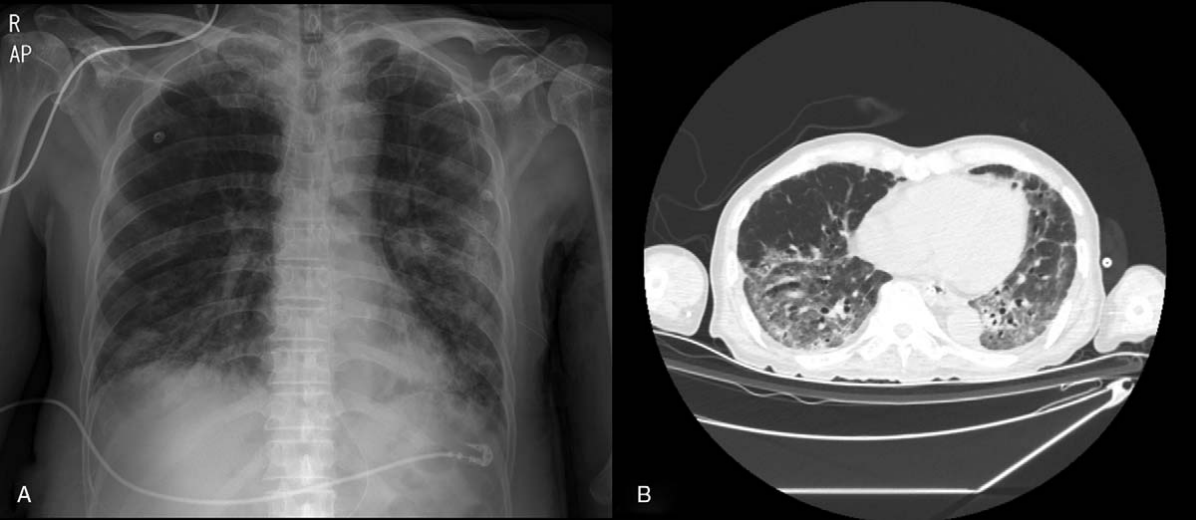
CXR and CT scan revealed diffuse viral pneumonia associated with COVID-19. A, CXR. B, CT scan. CXR = Chest X-ray.

A 74-year-old man with no previous medical history visited our hospital with complaints of fever and sore throat. A polymerase chain reaction (PCR) test confirmed that he had COVID-19, and he was hospitalized in a negative pressure isolation ward. He had difficulty breathing, which gradually worsened. Ten days after admission, he was intubated and mechanical ventilation began. Diffuse viral pneumonia was identified on chest radiography and computed tomography (Fig. 1). After a month of mechanical ventilation, he was weaned off of the ventilator. However, critical illness and prolonged treatment in the ICU resulted in severe muscle wasting and deterioration of physical function.

**Figure 2:**
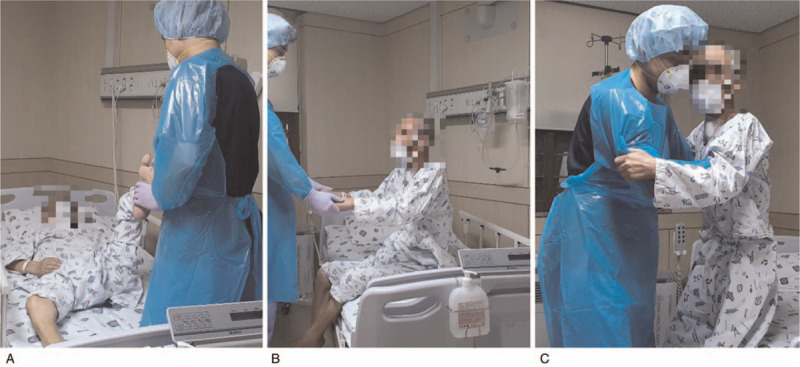
Step-by-step progress of rehabilitation tailored to functional recovery of the patient. A, Manual joint ROM exercise (step 1). B, Sitting balance training (step 2). C, Standing balance training (step 3) and sit-to-stand training (step 4).

For a comprehensive rehabilitation treatment, he was referred to the Department of Rehabilitation Medicine. During the initial assessment, he needed moderate support for static sitting balance and scored 0 on the Berg Balance Scale and 8 on the Modified Barthel Index. The strength of both the upper and lower limbs was grade 3 following the Medical Research Council (MRC) grade. He was clinically diagnosed as ICU-acquired weakness because the MRC sum sore consisting of both wrist extension, elbow flexion, shoulder abduction, ankle dorsiflexion, knee extension, and hip flexion was 36 points, which was less than 48 points suggested by the guideline.^[11]^ Various types of rehabilitation exercises were carried out in a step-by-step manner according to the patient's function (Fig. 2). The bed manual joint range of motion (ROM) exercise (step 1) and position change were performed six times daily for 10 minutes by a healthcare provider authorized to enter the quarantine ward while wearing Level D protective equipment. After two consecutive follow-up PCR tests were negative for COVID-19, the patient was transferred to the general ward. There, rehabilitation therapy was performed by a physical therapist and an occupational therapist. In addition to the ROM exercises previously performed, sitting balance training (step 2), upper and lower extremity strength exercises, and pulmonary rehabilitation were done. The pulmonary rehabilitation included inspiratory muscle strength training using an incentive spirometer, air stacking exercise, and airway secretion removal using a cough induction machine. The exercises were performed daily for 30 minutes on a one-to-one basis by the patient with the therapist in the hospital room. The therapist wore a working gown, disposable cap, latex gloves, and N95 mask as protective equipment, while the patient wore a mask. During the videofluoroscopic swallow study (VFSS) test for evaluation of dysphagia, aspiration was observed with both thick and thin fluids. Feeding through the Levin tube was continued, and the rehabilitative dysphagia treatment including chin tuck against resistance, thermotactile stimulation and electirical stimulation therapy was performed for 30 minutes daily.

Two weeks later, he was able to maintain sitting balance with supervision and static standing balance with moderate support. From this point on, the rehabilitation treatment consisted of standing balance training (step 3) and sit-to-stand training (step 4).

After a total of 4 weeks of treatment, the patient was transfered to the local hospital specialized in rehabilitation and continuously performed in the rehabilitation. In the evaluations just before transfer, the patient's Berg Balance Scale improved to 4 and Modified Barthel Index to 18. The MRC grade of both the upper and lower limbs improved to 3 plus. During the follow-up VFSS test, aspiration was observed only with thin fluid, and only penetration was observed with thick fluid, semisolid, and solid diets. Oral feeding was started with a soft diet and food thickener. During the pulmonary function test (PFT), forced vital capacity (FVC) was 1.19 L (31% of the predicted value), forced expiratory volume in 1 second as 1.14 L (31% of the predicted value), forced expiratory volume in one second 1/FVC was 96% and peak expiratory flow was 4.24 L/sec. The PFT test suggested a moderate restrictive lung defect based on the FVC. The PFT could not be performed at the time of initial evaluation due to the poor general condition of the patient and his inability to abey commands.

## 3 Discussion

In this case report, early rehabilitation in the isolation ward, inpatient rehabilitation after negative PCR tests, and respiratory rehabilitation were administered to the patient from the time of admission to the ICU until transfer to the general ward.

Early rehabilitation for critically ill patients in the ICU is important and feasible.^[12]^ Early physical and pulmonary rehabilitation treatment can reduce the duration of ventilator dependence, hospitalization, and treatment in the ICU, and can improve muscle strength, exercise capacity and walking capacity.^[13,14]^ Rehabilitation in the ICU can be performed relatively safely with a low risk of potential adverse events, even if the patients in the ICU receive ventilator support, continuous renal replacement therapy, or extracorporeal membrane oxygenation.^[15–17]^ ICU- acquired weakness occurs in a large number of COVID-19 patients severe enough to require ICU care. Therefore, the importance of early rehabilitation after COVID-19 is being emphasized. Yu et al argued that early rehabilitation for critically ill patients with COVID-19 through the cooperation of multiple teams would have more benefits than risks.^[18]^ Levy et al presented a model of unit for ventilator weaning and early rehabilitation in COVID-19 patients.^[19]^ And, Curci et al proposed an early rehabilitation protocol for post-acute COVID-19 based on FiO2 levels.^[20]^

Although early rehabilitation treatment is effective for COVID-19 patients, the safety of therapists is a new topic because of the highly contagious nature of COVID-19.^[10]^ If the isolated patient is conscious, rehabilitation through remote consultation or non-face-to-face education using a video or booklet is recommended.^[21]^ In patients with reduced levels of consciousness and difficulties with coordination, it is recommended that medical personnel entering the isolation ward perform basic joint ROM exercises and strength exercises. In this case, ROM exercises were performed by the ICU medical staff because the patient was unable to cooperate due to the use of a sedative agent to reduce fighting during ventilator treatment.

After the negative COVID-19 PCR tests and transfer to the general ward, rehabilitation treatment was performed by rehabilitation therapists. Disposable caps, medical face masks, latex gloves, working gowns, and hand sanitizers are recommended for protection during rehabilitation treatment of patients who are negative and do not generate aerosol.^[21]^ The therapists wore all the recommended equipment during rehabilitation treatment. The rehabilitation treatment for this patient did not advance further from standing balance and sit-to-stand trainings, but walking and aerobic trainings using an ergometer or treadmill could be performed if the patient had better function.^[22]^

In the early days following transfer to the general ward, the patient needed oxygen supply, so pulmonary rehabilitation was also performed. At the time of discharge, breathing was possible without an oxygen supply. Although there was no oxygen demand, PFT to evaluate the effectiveness of respiratory rehabilitation identified moderately restrictive lung disease due to permanent lung injury, a sequelae of COVID-19.^[23]^

## 4 Conclusion

This case report shows that rehabilitation treatment for patients with COVID-19 is effective for functional recovery and can be safely performed from the ICU to the general ward with appropriate protective gear and prevention policies.

## Author contributions

Conceptualization, J.H.; writing—original draft preparation, D.G.; writing—review and editing, J.H. All authors contributed to the article and approved the submitted version.

**Conceptualization:** Jong-Moon Hwang.

**Writing – original draft:** Dae-Won Gwak.

**Writing – review & editing:** Jong-Moon Hwang.
